# The Functions of Pt-DIC and Pt-Lamin B in Spermatogenesis of *Portunus trituberculatus*

**DOI:** 10.3390/ijms25010112

**Published:** 2023-12-21

**Authors:** Shuo-Yue Wang, Qiu-Meng Xiang, Jun-Quan Zhu, Chang-Kao Mu, Chun-Lin Wang, Cong-Cong Hou

**Affiliations:** Key Laboratory of Aquacultural Biotechnology, Key Laboratory of Marine Biotechnology of Zhejiang Province, College of Marine Sciences, Ningbo University, Ningbo 315211, China; 2111091164@nbu.edu.cn (S.-Y.W.); 1911091064@nbu.edu.cn (Q.-M.X.); zhujunquan@nbu.edu.cn (J.-Q.Z.); muchangkao@nbu.edu.cn (C.-K.M.); wangchunlin@nbu.edu.cn (C.-L.W.)

**Keywords:** crustacean, gametogenesis, maturation, cellular localization, expression analysis

## Abstract

Cytoplasmic Dynein is a multiple-subunit macromolecular motor protein involved in the transport process of cells. The Dynein intermediate chain (DIC) is one of the subunits of Dynein-1. In our previous studies, we showed that Pt-DIC may play an important role in the nuclear deformation of spermiogenesis in *Portunus trituberculatus*. Lamin B is essential for maintaining nuclear structure and functions. Surprisingly, Pt-Lamin B was expressed not only in the perinucleus but also in the pro-acrosome during spermiogenesis in *P. trituberculatus*. Studies have also shown that Dynein-1 can mediate the transport of Lamin B in mammals. Thus, to study the relationship of Pt-DIC and Pt-Lamin B in the spermatogenesis of *P. trituberculatus*, we knocked down the *Pt-DIC* gene in *P. trituberculatus* by RNAi. The results showed that the distribution of Pt-DIC and Pt-Lamin B in spermiogenesis was abnormal, and the colocalization was weakened. Moreover, we verified the interaction of Pt-DIC and Pt-Lamin B via coimmunoprecipitation. Therefore, our results suggested that both Pt-DIC and Pt-Lamin B were involved in the spermatogenesis of *P. trituberculatus*, and one of the functions of Dynein-1 is to mediate the transport of Lamin B in the spermiogenesis of *P. trituberculatus*.

## 1. Introduction

Spermatogenesis is a biological process involving spermatogonia division and differentiation and sperm formation. It is regulated by cytokines, hormones, and several genes. This process includes spermatogonial mitosis, spermatocyte meiosis, and spermiogenesis [1,2]. Mammalian spermiogenesis mainly includes acrosome formation, nucleus shaping and tail formation, and the formation of mature sperm with a head and tail [3]. Although the spermatogenesis process and regulatory mechanism of different species are similar, there are great differences in sperm morphology and chromatin composition [4]. The spermatogenesis process of decapod crustaceans is similar to that of mammals [5]. However, the sperm formation of decapod crustaceans lacks the process of tail formation, and the sperm nuclei of mature sperm are unconcentrated [4,5]. *Portunus trituberculatus* is an important marine economic crab in China. The basic reproductive biology of *P. trituberculatus* is a hot research field. The spermatozoa of *P. trituberculatus* has the typical non-concentrated nucleus of decapod crustaceans [4], which is suitable for studying molecular mechanisms, such as the nuclear deformation of spermatids. Therefore, it is an ideal model to study the molecular mechanisms underlying reproductive biology in decapod crustaceans.

Sperm nuclear morphology is one of the substantial indicators used to assess sperm quality, which is crucial for the fertilization process [6,7]. Nuclear structures undergo remarkable changes during spermatogenesis from spermatogonia to mature sperm [8]. The nuclear skeleton and motor proteins are essential for maintaining the function of the nucleus [9,10]. The nuclear lamina is a network structure composed of Lamins, which are located on the inner side of the nuclear membrane [11]. Lamins are an evolutionarily highly conserved V-type intermediate filament (IF) protein superfamily whose functions are maintaining the shape and size of the nucleus, nuclear localization, DNA replication, and DNA damage repair [12,13,14,15]. Lamin B is very important for maintaining the function of nuclear morphology [16]. Lamins can maintain the integrity of the nuclear envelope by forming a network structure within the nucleus [17]. Two Lamins can form a dimer through the α-helical central rod structural domain of the spiral coil [17]. Two or more Lamin dimers can form Lamin polymers by connecting their heads and tails in parallel [17,18,19]. These Lamin polymers form Lamin tetramers through reverse parallel interactions, and each Lamin can form a unique network structure [19].

Knockout or mutation of Lamins can lead to changes in nuclear morphology [11,20]. Lamins are components of the nuclear lamina (NL), where Lamin A, B1, and B2 proteins can be recruited to the inner nuclear membrane (INM) via their own nuclear localization signals (NLS) [21]. Lamins are classified into A and B types according to their protein structure and characteristics [21]. Mammalian type A Lamins include Lamin A, A∆10, C, and C2, which are all encoded by *LMNA*; type B Lamins include Lamin B1, which is encoded by *LMNB1*; and Lamin B2 and B3, which are encoded by *LMNB2* [19,21]. Type A Lamins mainly play a role in cell differentiation, and their expression is related to development [22]; type B Lamins are widely expressed in somatic cells, while Lamin C2/B3 is only present in germ cells [22,23,24]. In mammals, Lamin B1 is usually expressed only in proliferating epithelial cells [25]. Lamin B2 is commonly expressed at all stages of spermatogenesis, and Lamin B3 is only expressed at a specific stage in spermatogenesis [25,26]. At least one type of B Lamin is expressed in invertebrate cells [27]. Type B Lamin (Dm) in *Drosophila melanogaster* is similar to mammalian type B Lamin and is widely expressed throughout development [27,28]. *Caenorhabditis elegans* contains only one Lamin gene (*Lmn-1*), which is a type B Lamin and is widely expressed in all cells except mature sperm [27,29]. The loss of function of Lamin B3, which is specifically expressed in mouse spermatocytes, leads to a change in the shape of the nucleus from spherical to hooked [30]. In *D. melanogaster*, the NL composed of Lamin Dm is continuously reorganized in the early stage of meiosis of spermatogenesis until the end of spermatogenesis [8]. Lamin B1 is involved in the fragmentation process of the nuclear membrane during mitosis and participates in the reorganization of the nuclear periphery after mitosis [31,32]. Lamin B1 can be used as an autophagy substrate in human fibroblasts expressed by an oncogene (HRasV12), leading to its autophagy degradation in the cytoplasm through the transport process from the nucleus to the cytoplasm [33,34,35]. The above studies indicate that Lamin B maintains nuclear morphology and function during spermatogenesis. However, there are few studies on the function of Lamin B in the process of spermiogenesis in crustaceans.

Nuclear deformation is associated with the nuclear skeleton and motor proteins during spermiogenesis [5,8,26]. Motor proteins are considered to be the main driving force for cell material transport and deformation [36]. Dynein is a multiple-subunit complex motor protein that is built around the core of the large dimeric Dynein heavy chain (DHC), transporting organelles via adenosine triphosphate (ATP) hydrolysis and assembling the Dynein intermediate chain (DIC), Dynein light intermediate chain (DLIC), and Dynein light chain (DLC) into a Dynein-derived large complex [37,38]. Dynein includes axoneme Dynein and cytoplasmic Dynein [37]. The former exists in cell cilia and sperm flagella and participates in cell movement, while the latter can transport intracellular organelles to the negative end of microtubules [37]. Cytoplasmic Dynein contains two subtypes. One is cytoplasmic Dynein 2 (Dynein-2), which exists in flagella or cilia and participates in intracellular transport (IFT) [37]. The other is cytoplasmic Dynein 1 (Dynein-1), which transports goods along microtubules to the negative end in the cytoplasm [37]. In Dynein, different parts of the components possess different functions [39]. Among them, DIC, as a part of Dynein, exists as a dimer in Dynein and plays a supporting role, which can bind with DHC, DLIC, DLC, and some Dynein regulators (e.g., NudE, dynactin, etc.) [37,39]. When the heavy chain of cytoplasmic Dynein 1 (*Dync1h1*) is knocked out in mice, the formation of the blood–testis barrier is hindered [40]. When *Tcte3-3*, a dynamin light chain gene in mouse testes, was defective, sperm motility was reduced [41]. Mutations in the *D. melanogaster* Dynein intermediate chain gene *DIC61B* can cause abnormal sperm morphology [42]. During spermatogenesis in *P. trituberculatus*, Dynein can participate in the process of nuclear morphology and acrosome formation by transporting organelles such as mitochondria and the Golgi apparatus [5]. These studies showed that Dynein-1 plays a crucial role in spermiogenesis.

Based on the above studies, we propose the following hypothesis: the type B Lamin and DIC from *P. trituberculatus*, Pt-Lamin B, and Pt-DIC may be involved in the spermiogenesis process of the crab, and there may be an interaction between them. To verify our hypothesis, we cloned the full-length fragment of *Pt-Lamin B* and analyzed its sequence and protein structure. As Dynein-1 is a complex with a large molecular weight and a complex structure, it is difficult to study as a whole. Therefore, we chose DIC, an essential subunit of Dynein-1, as the research object. We preliminarily studied the distribution and localization of Pt-DIC and Pt-Lamin B in spermatogenesis via immunofluorescence. We also explored the interaction between Pt-DIC and Pt-Lamin B via immunoprecipitation. To further explore the function of Pt-DIC, we knocked down the expression of *Pt*-*DIC* using RNAi and studied the expression and distribution characteristics of the Dynein-1 and Pt-Lamin B genes and proteins via quantitative and immunofluorescence methods, as well as their effects on the spermatogenesis of *P. trituberculatus*. This study provides a theoretical basis for revealing the mechanism of spermatogenesis in decapod crustaceans.

## 2. Results

### 2.1. Prediction of the Full-Length Sequence of Pt-Lamin B cDNA and Its Protein Structure

**Figure 1:**
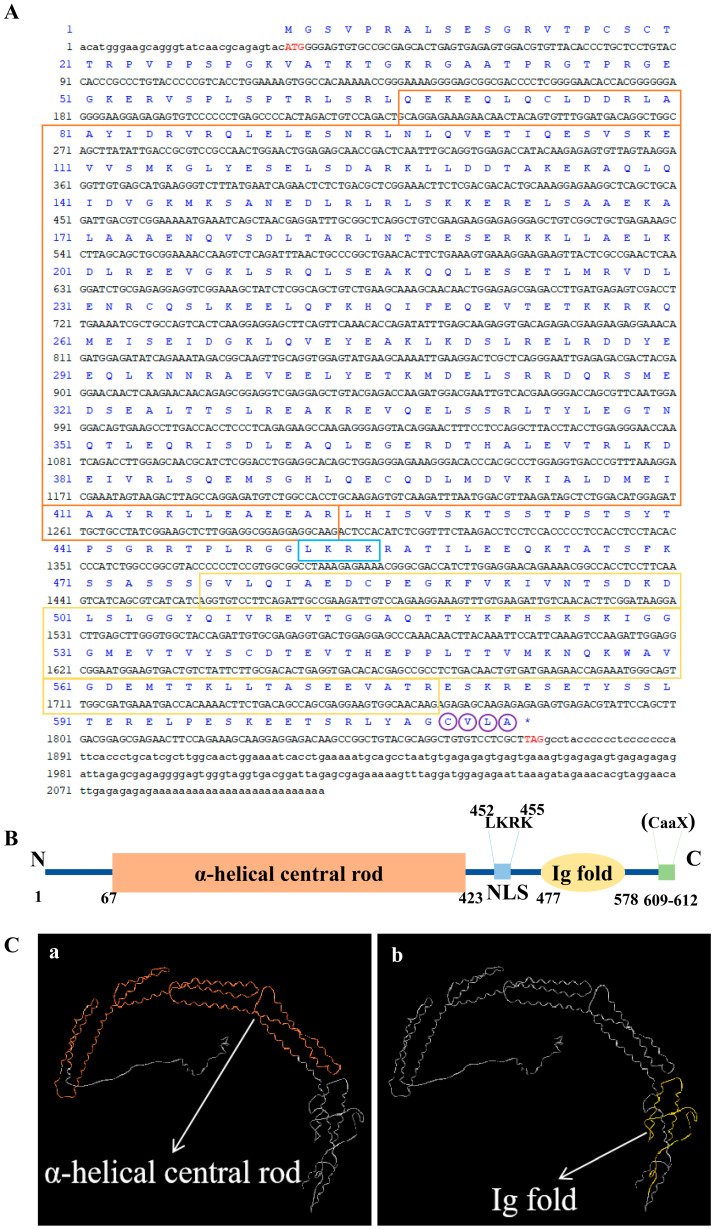
The full-length sequence of *Pt-Lamin B* gene and the secondary and tertiary structures of Pt-Lamin B protein. (**A**) The base sequence of the red font is the start and terminal codons. The sequence in the vermilion box is the α-helical central rod domain. The sequence in the yellow boxes is the Ig fold domain, the amino acid sequence in the blue box is the NLS, and the amino acid sequence in the purple circles is the CaaX motif unique to the IF protein family. “*” indicates a terminal codon. (**B**) The secondary structure of Pt-Lamin B protein. The orange region is the α-helical central rod domain (aa: 67–423), the yellow region is the Ig fold domain (aa: 477–578), and the blue region is the NLS (aa: 452–455). The C-terminal region had a CaaX motif unique to the IF protein family (aa: 609–612). (**C**) In the tertiary structure of Pt-Lamin B protein(a, b), the orange region of figure a is the α-helix central rod domain, and the yellow region of figure b is the Ig fold domain.

The *Pt-Lamin B* sequence was uploaded to NCBI (GenBank: OQ506130). The total length of *Pt-Lamin B* cDNA was 2107 bp, including 31 bp of the 5′ untranslated region (UTR), 237 bp of the 3′ UTR, and 1839 bp of the open reading frame, encoding 612 amino acids (Figure 1A).

We analyzed the properties and structures of the Pt-Lamin B protein. We used online tools to predict the molecular weight of Pt-Lamin B to be approximately 68.98 kDa, with an isoelectric point of approximately 5.56. According to online tool predictions of the secondary and tertiary structures of the Pt-Lamin B protein, Pt-Lamin B contains an N-terminal head domain, an IF protein domain, a Lamin tail domain containing an Ig fold, an NLS, and a CaaX motif (C, cysteine; a, aliphatic residue; X, any residue) at the C-terminus (Figure 1B,C).

### 2.2. Pt-Lamin B Multiple Sequence Alignment and Phylogenetic Tree Analysis

**Figure 2:**
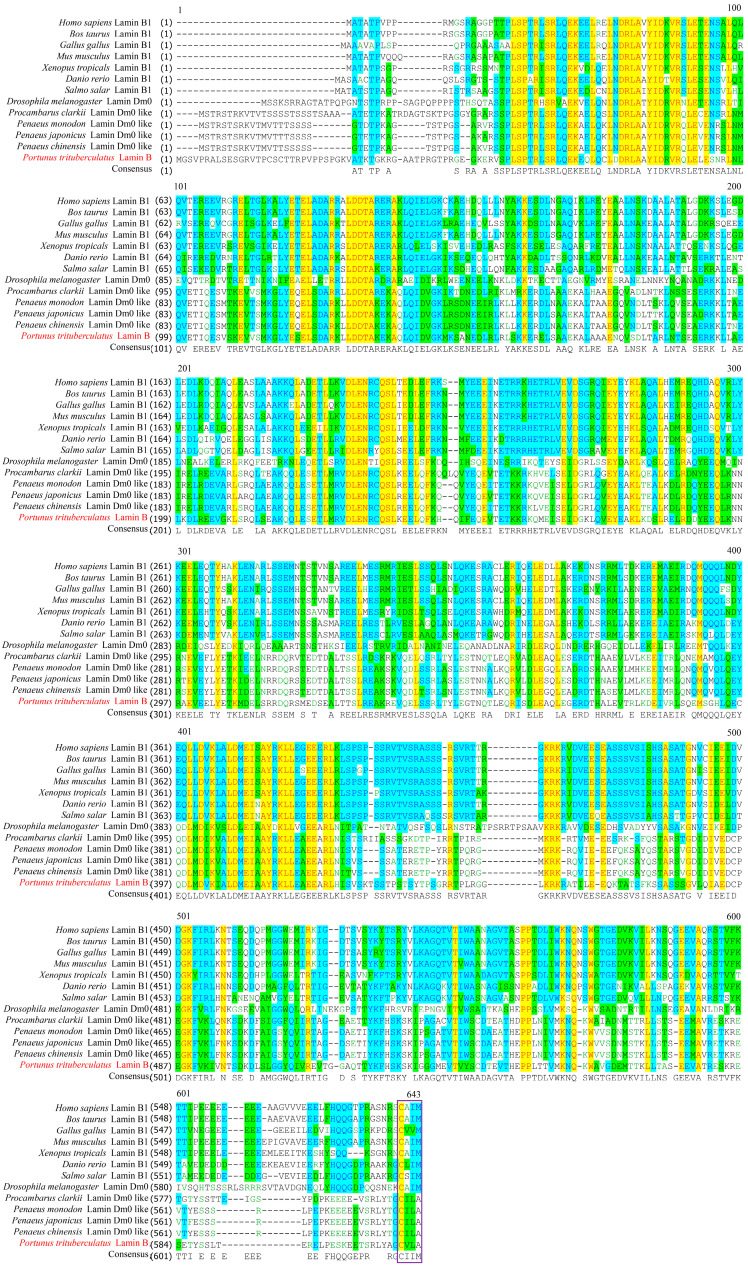
Multiple alignment of Pt-Lamin B with homologous sequences from vertebrate and invertebrate species. The same amino acids (aa) are indicated by yellow shading. The light blue shading indicates areas with 0.50% similarity and green shading represents areas with lower similarity. The red font is the amino acid sequence of the Lamin B protein of *P. trituberculatus.* The purple-boxed amino acid sequences are CaaX motifs unique to the IF protein family.

The amino acid sequence alignment revealed the similarity of *P. trituberculatus* Lamin B with other species, and both had CaaX motifs unique to the IF protein family (Figure 2). The results of the amino acid sequence comparison of various species of Lamin B showed that Pt-Lamin B had high similarity with crustacean Lamin B; the highest similarity occurred between *P. trituberculatus* and *Procambarus clarkii*, reaching 66.1%. It was followed by *Penaeus japonicus* (64.2% identity), *Penaeus monodon* (63.7% identity), and *Penaeus chinensis* (63.7% identity), but low similarity with vertebrate Lamin B (Table 1).

**Figure 3:**
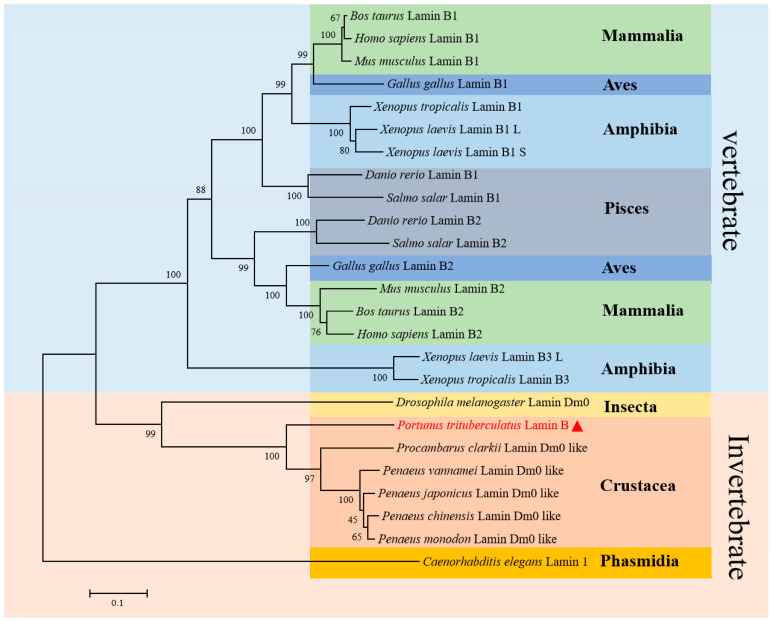
Phylogenetic tree analysis of Lamin B. Pt-Lamin B of *P. trituberculatus* shows the closest evolutionary relationship with Decapoda but a more distant evolutionary relationship with Lamin B vertebrates. The largest light blue part is for vertebrates, and the largest orange part is for invertebrates. The green regions are Mammalia. The dark blue regions are Aves and Amphibia. The light blue regions are Amphibia. The red region is Crustacea. The yellow regions are Insecta. The orange region is Phasmidia. The gray region is Pisces. The red triangle and red font are *P. trituberculatus*.

Phylogenetic analysis of Lamin B amino acid sequences showed that Pt-Lamin B clustered with other crustacean Lamin B with high support and was thus sister to other invertebrate Lamin B (Figure 3).

### 2.3. Validation of the Pt-Lamin B Antibody

**Figure 4:**
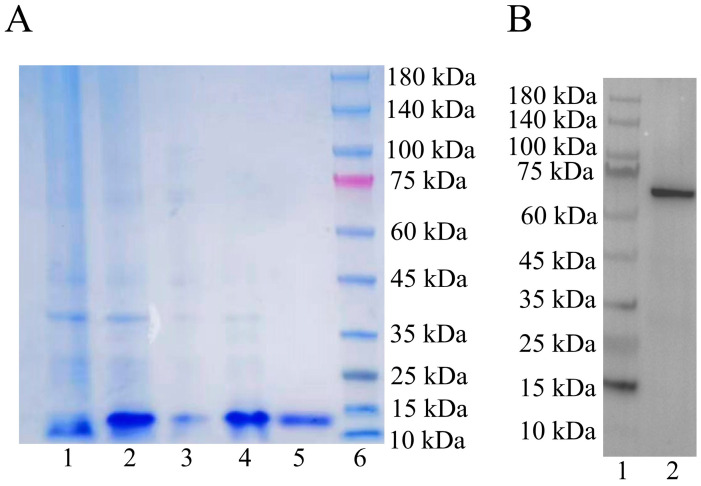
Expression of the Pt-Lamin B recombinant protein and rabbit anti-Pt-Lamin B antibody-specificity detection. (**A**) Expression of Pt-Lamin B recombinant protein. Line 1: uninduced bacterial liquid; Line 2: IPTG-induced bacterial fluid for 6 h; Line 3: supernatant of recombinant protein expression of Pt-Lamin B; Line 4: sediment of recombinant protein expression of Pt-Lamin B; Line 5: purified Pt-Lamin B recombinant protein; Line 6: maker. (**B**) Rabbit anti-Pt-Lamin B antibody. The results showed that the Pt-Lamin B bands were single and consistent with the predicted size of Pt-Lamin B (68.98 kDa). Line 1: Protein marker; Line 2: Pt-Lamin B protein band.

We validated the specificity of the Pt-Lamin B antibody using Western blot technology. The protein band was single, consistent with the predicted size of the Pt-Lamin B protein of 68.98 kDa (Figure 4B).

### 2.4. Expression and Distribution of Pt-Lamin B during Spermatogenesis in P. trituberculatus

**Figure 5:**
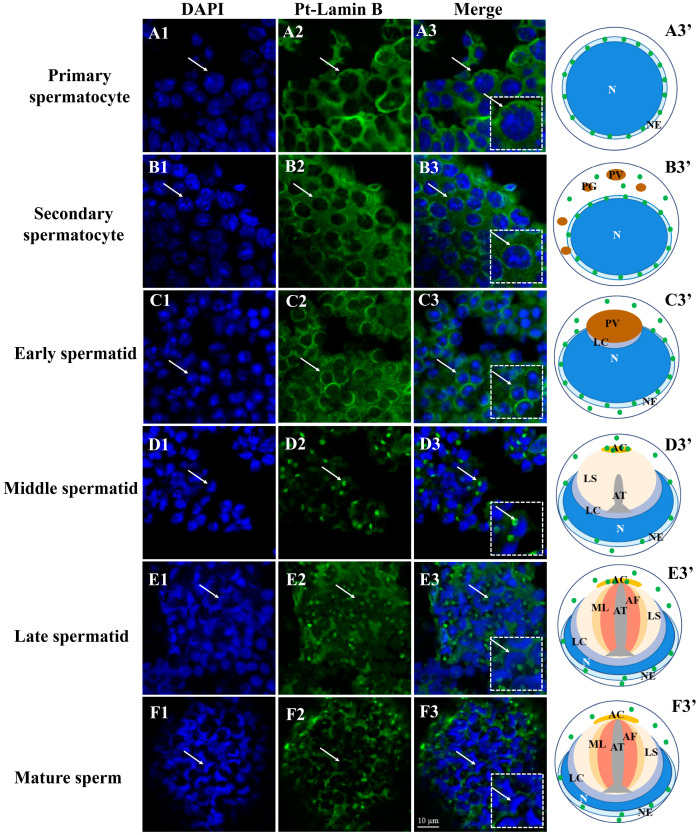
Distribution of Pt-Lamin B during spermatogenesis in *P. trituberculatus.* The blue signal represents the nucleus, the green signal represents Pt-Lamin B, and the white arrow points to the spermatid at different stages. The white dotted boxes represent enlarged images of spermatids pointed by white arrows. The white arrows in the dotted white box represent enlarged spermatids. In the pattern map (**A3′**,**B3′**,**C3′**,**D3′**,**E3′**,**F3′**), the green dots represent the signal distribution of Pt-Lamin B in spermatids at different stages of spermatogenesis. (**A1**–**A3**′) primary spermatocytes; (**B1**–**B3**′) secondary spermatocytes; and (**C1**–**C3**′) early spermatids, Lamin B was distributed in the nuclear membrane and in the cytoplasm. (**D1**–**D3**′) middle spermatids. (**E1**–**E3**′) Pt-Lamin B also had a strong signal in the acrosome complex in late spermatids. In (**F1**–**F3**′) mature sperm, Pt-Lamin B was still distributed in the perinuclear region and cytoplasm. However, the signal in the acrosome was weakened. The right column of (**A3′**,**B3′**,**C3′**,**D3′**,**E3′**,**F3′**) is a pattern map drawn based on the Pt-Lamin B position. N: nuclear; PG: proacrosomal granules; PV: proacrosomal vesicle; LC: layered complex; AC: acrosome cap; AT: acrosome tube; AF: acrosome fiber; ML: middle layer; LS: layered structure; NE: nuclear envelope. Scales = 10 μm.

Using immunofluorescence, we detected Pt-Lamin B expression and distribution during spermatogenesis in *P. trituberculatus* (Figure 5). In primary spermatocytes (Figure 5A1–A3′), Pt-Lamin B was clearly localized on the nuclear membrane. In secondary spermatocytes (Figure 5B1–B3′) and early spermatids (Figure 5C1–C3′), Pt-Lamin B was localized on the nuclear membrane and the cytoplasm. In the middle spermatids (Figure 5D1–D3′) and late spermatids (Figure 5E1–E3′), Pt-Lamin B was localized on the nuclear membrane and cytoplasm and had a strong signal on the gradually formed acrosome. In mature sperm (Figure 5F1–F3′), the Pt-Lamin B signals still existed in the nuclear membrane and cytoplasm, but the signals in the acrosome were weakened.

### 2.5. Colocalization of Pt-DIC and Pt-Lamin B during Spermiogenesis in P. trituberculatus

**Figure 6:**
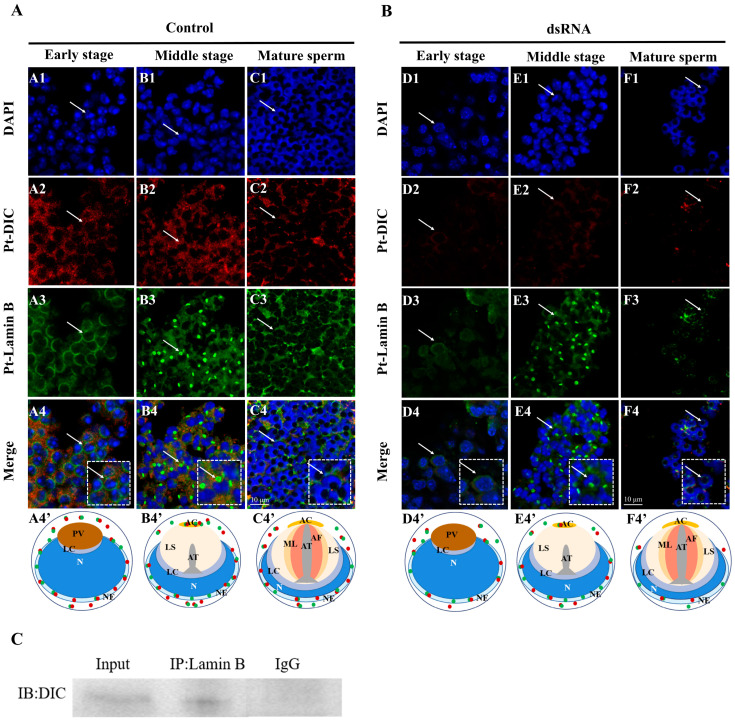
Expression distribution, co-localization, and immunoprecipitation of Pt-Lamin B and Pt-DIC during the spermatogenesis of *P. trituberculatus* after interference of the *Pt-DIC* gene. The white dotted boxes represent enlarged images of spermatids pointed by white arrows. The white arrows in the dotted white box represent enlarged spermatids. In the pattern map (A4′,B4′,C4′,D4′,E4′,F4′), the green dots represent the signal distribution of Pt-Lamin B in spermatids at different stages of spermiogenesis; The red dots represent the signal distribution of Pt-DIC in spermatids at different stages of spermiogenesis. (**A**) PBS control group: Blue signal represents the nucleus, red signal represents Pt-DIC, green signal represents Pt-Lamin B, and white arrows point to spermatids at different stages. In early spermatids (A1–A4′), both Pt-DIC and Pt-Lamin B were localized around the nucleus and in the cytoplasm. In the middle spermatids (B1–B4′), Pt-DIC and Pt-Lamin B were still colocalized around the nucleus and in the cytoplasm. Pt-DIC and Pt-Lamin B were also colocalized in the acrosome. In the mature sperm (C1–C4′), Pt-DIC and Pt-Lamin B were still colocalized around the nucleus and in the cytoplasm. The signals of Pt-DIC, its colocalization with Pt-Lamin B, and the signal of both in the acrosome were weakened. The last row of A4′, B4′, and C4′ is a pattern map drawn according to the positions of Pt-Lamin B and Pt-DIC. Scales = 10 μm. N: nuclear; PG: proacrosomal granules; PV: proacrosomal vesicle; LC: layered complex; AC: acrosome cap; AT: acrosome tube; AF: acrosome fiber; ML: middle layer; LS: layered structure; NE: nuclear envelope. (**B**) ds-*DIC*-RNA experimental group: Blue signal represents the nucleus, red signal represents Pt-DIC, and white arrows point to sperm cells at different stages. Colocalization of Pt-Lamin B and Pt-DIC weakened in early spermatids (D1–D4′), middle spermatids (E1–F4′), and mature sperm (F1–F4′). The last line of D4′, E4′, and F4′ is a pattern map based on Pt-Lamin B and Pt-DIC positions. Scales = 10 μm. N: nuclear; PG: proacrosomal granules; PV: proacrosomal vesicle; LC: layered complex; AC: acrosome cap; AT: acrosome tube; AF: acrosome fiber; ML: middle layer; LS: layered structure; NE: nuclear envelope. (**C**) Detection of the interaction between Pt-DIC and Pt-Lamin B via co-immunoprecipitation. IB: immunoblotting; Input: positive control; protein samples before IP; IP: protein sample of immunoprecipitation; IgG: negative control, rat serum immunoprecipitation product without immune antigen.

In early spermatids (Figure 6A (A1–A4′)), both Pt-DIC and Pt-Lamin B were localized around the nucleus and in the cytoplasm. In the middle spermatids (Figure 6A (B1–B4′)), Pt-DIC and Pt-Lamin B were still colocalized around the nucleus and in the cytoplasm. Pt-DIC and Pt-Lamin B were also colocalized in the acrosome. In the mature sperm (Figure 6A (C1–C4′)), Pt-DIC and Pt-Lamin B were still colocalized around the nucleus and in the cytoplasm. The signals of Pt-DIC, its colocalization with Pt-Lamin B, and the signal of both in the acrosome were weakened.

### 2.6. Effects of Interference with Pt-DIC at the Individual Level on Spermatid Nucleus Deformation and Spermiogenesis

#### 2.6.1. Preparation of *Pt-DIC* Gene dsRNA

**Figure 7:**
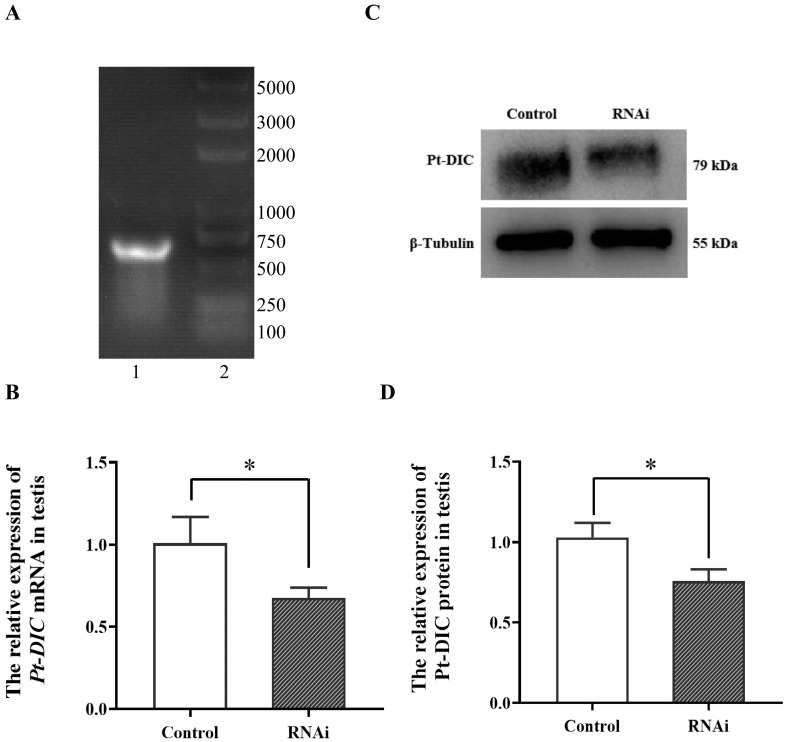
Detection of gene and protein expression after in vivo interference with *Pt-DIC* in *P. trituberculatus.* (**A**) Synthesized *Pt-DIC*-specific dsRNA. Lane 1: purified ds-*DIC*-RNA bands, Line 2: marker, unit: bp. (**B**) qPCR was used to detect the expression of *Pt-DIC* mRNA in the testes of *P. trituberculatus*. After dsRNA interference, the relative expression level of *Pt-DIC* mRNA significantly decreased. (**C**) Western blot was used to detect the expression of Pt-DIC protein in the testes of *P. trituberculatus*. The Pt-DIC protein bands became weaker after dsRNA interference compared to the control group. (**D**) The gray level values extracted from C were analyzed using ImageJ software (http://rsb.info.nih.gov/ij/index.html). The expression level of Pt-DIC protein significantly decreased compared to that in the control group after interference; ‘*’ indicates *p* < 0.05.

To further study the function of Pt-DIC in the spermiogenesis of *P. trituberculatus*, we designed a 464 bp *Pt-DIC* gene fragment and prepared *Pt-DIC* dsRNA with this fragment as a template through a TranscriptAid T7 high-yield transcription kit. The dsRNA bands were consistent with the expected size determined by gel electrophoresis (Figure 7A).

#### 2.6.2. Detection of Related Genes after In Vivo Interference of *Pt-DIC* Gene dsRNA

We injected the prepared *Pt-DIC*-dsRNA into *P. trituberculatus* five times in vivo, with an interval of 48 h each time. Then, we dissected each tissue on ice and extracted RNA and total protein from the testes tissue. After reverse transcription, the interference efficiency was detected by qPCR. The expression level of *Pt-DIC* mRNA significantly decreased after in vivo interference, with an interference efficiency of 32%. The interference effect was significant (Figure 7B). We performed Western blot detection on the extracted testes protein. The Pt-DIC protein bands in the interference group were weaker than those in the control group (Figure 7C). ImageJ was used to extract grayscale values for analysis. The results showed that the expression level of Pt-DIC protein significantly decreased after in vivo interference (Figure 7D).

**Figure 8:**
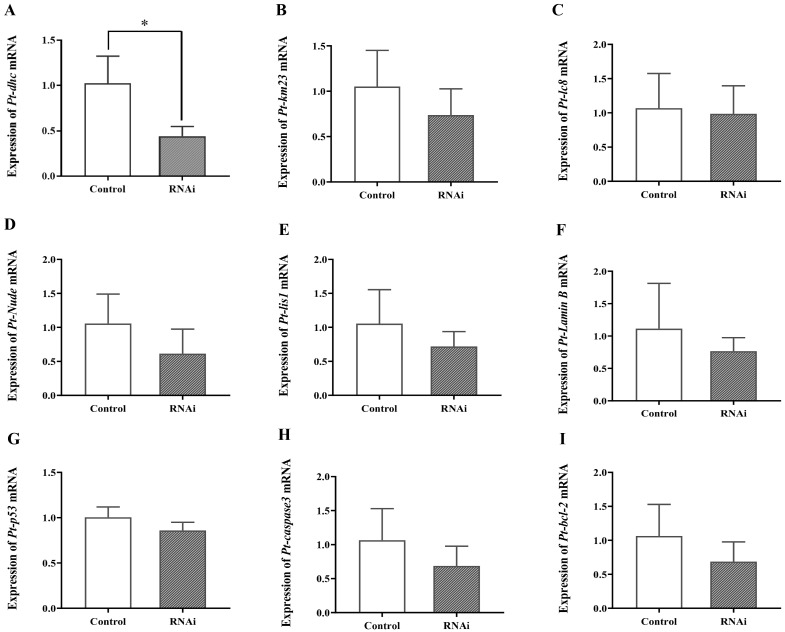
Detection of related genes after *Pt-DIC* in vivo interference. (**A**–**I**) represent the expression levels of *Pt-dhc*, *Pt-km23*, *Pt-lc8*, *Pt-Nude*, *Pt-Lis1*, *Pt-Lamin B*, *Pt-p53*, *Pt-caspase3*, and *Pt-bcl2* mRNA in the testes of *P. trituberculatus* after in vivo interference with *Pt-DIC*. ‘*’ indicates *p* < 0.05.

To study the effect of interfering with the expression of *Pt-DIC* on the testes of *P. trituberculatus*, the expression levels of six DIC-related and three apoptosis-related genes were detected by qPCR (Figure 8). After the decrease in the expression of *Pt-DIC*, the mRNA expression of the Dynein heavy chain *Pt-dhc* gene significantly decreased (Figure 8A). The expression of the Dynein light chain *Pt-km23* (Figure 8B) and *Pt-Lc8* (Figure 8C) genes and the Dynein-related regulatory factors *Pt-Nude* (Figure 8D) and *Pt-lis1* (Figure 8E) was not significantly changed. To investigate whether interference with *Pt-DIC* expression can lead to apoptosis in testes tissue, we also detected the expression of the apoptosis-related genes *Pt-p53* (Figure 8G), *Pt-caspase3* (Figure 8H), and *Pt-bcl2* (Figure 8I). There was no significant change in apoptosis-related genes.

#### 2.6.3. The Deformation of Spermatids Was Blocked after dsRNA Interference with the *Pt-DIC* Gene In Vivo

**Figure 9:**
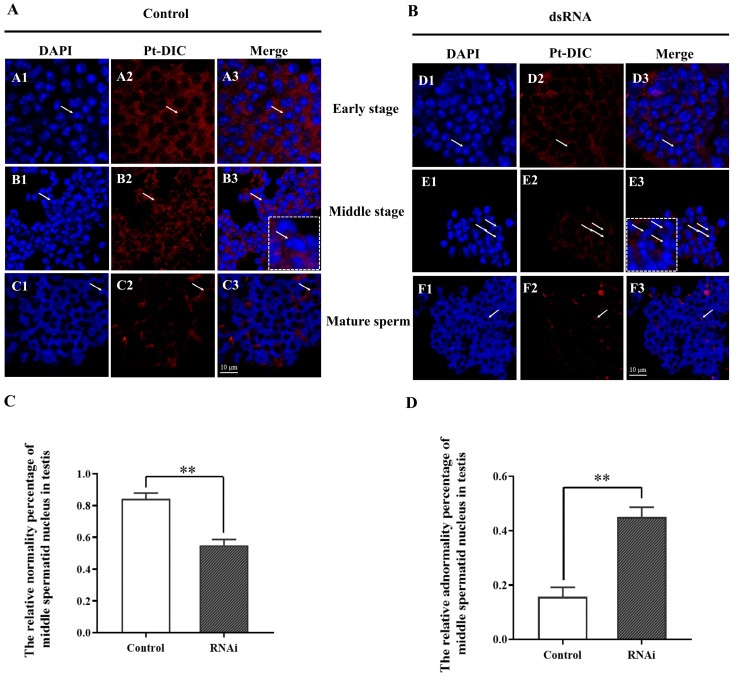
Changes in the distribution of Pt-DIC expression during spermatogenesis after in vivo interference with *Pt-DIC*. After interfering with *Pt-DIC* in vivo, the Pt-DIC signal decreased, and the nuclear morphology of spermatids in the middle stage was significantly abnormal. The blue signal represents the nucleus, the red signal represents Pt-DIC, and the white arrow points to the spermatids at different stages. The white dotted boxes represent enlarged images of spermatids pointed by white arrows. The white arrows in the dotted white box represent enlarged spermatids. (**A**) PBS control group, A1–A3: early spermatids; B1–B3: middle spermatids; C1–C3: mature sperm. (**B**) Injection of ds-*DIC*-RNA experimental group, D1–D3: early spermatids; E1–E3: middle spermatids; F1–F3: mature sperm. Scales = 10 μm. (**C**) The normal percentage of spermatid nuclei in the middle stage before and after in vivo interference with *Pt-DIC*; ‘**’ indicates *p* < 0.01. (**D**) The abnormal percentage of spermatid nuclei in the middle stage before and after in vivo interference with *Pt-DIC*; ‘**’ indicates *p* < 0.01.

The Pt-DIC signal was significantly weakened in the early spermatids (Figure 9B (D1–D3)) of the interference group compared to the early spermatids (Figure 9A (A1–A3)) of the control group. In the middle spermatids, the percentage of normal nuclear deformation of spermatids in the interference group was significantly lower than that in the control group (Figure 9C), and the percentage of abnormal nuclear deformation of spermatids in the interference group was significantly higher than that in the control group (Figure 9D), while the Pt-DIC signal was weakened (Figure 9B). In mature sperm, no significant difference was observed between the interference group (Figure 9B (F1–F3)) and the control group (Figure 9A (C1–C3)).

### 2.7. Changes in the Distribution of Pt-Lamin B after In Vivo Interference with the Pt-DIC Gene dsRNA

The expression of Pt-DIC in early spermatids (Figure 6B (D1–D4′)) of the interference group decreased compared to that of the control group (Figure 6A (A1–A4′)), and the colocalization of Pt-Lamin B and Pt-DIC decreased. After interference with Pt-DIC, the colocalization of the Pt-Lamin B signal and Pt-DIC in middle spermatids (Figure 6B (E1–E4′)) decreased compared to that in the control group (Figure 6A (B1–B4′)). In mature sperm, there was no significant difference between the Pt-Lamin B signal and Pt-DIC signal after interference with Pt-DIC (Figure 6B (F1–F4′)) and before interference (Figure 6A (C1–C4′)).

### 2.8. Analysis of the Interaction between the Pt-DIC Protein and Pt-Lamin B Protein

We used Pt-DIC and Pt-Lamin B antibodies for immunoprecipitation and found that Pt-DIC protein interacted with Pt-Lamin B protein in the *P. trituberculatus* testes (Figure 6C).

## 3. Discussion

### 3.1. Protein Structural Characteristics of Pt-Lamin B

Lamin B, as a member of the IF family, has the common structural characteristics of the IF family [43]. It includes an N-terminal head domain, an α-helical central rod domain of the spiral coil, and a C-terminal tail domain [43]. The unique tail domain of the IF protein family contains an NLS, an Ig fold domain, and a CaaX motif [14,43]. The Ig-fold domain is the key to the interaction between Lamin and other proteins and chromatin binding [43,44]. The CaaX motif plays an important role in the process of Lamin anchoring to the inner nuclear membrane [43,45]. The Pt-Lamin B protein structure predicted in this study has the common characteristics of the IF family. Pt-Lamin B is similar to the Lamin B structure in other species. We speculate that Pt-Lamin B also performs the same functions as the intermediate filamentin family through its own domain and is involved in maintaining nuclear integrity in the form of Lamin polymers.

### 3.2. Lamin B Is Involved in Nuclear Deformation and Acrosome Formation

In the process of spermiogenesis, the spermatid nucleus undergoes chromatin remodeling, which makes the spermatid nucleus undergo great changes [46]. The nuclear layer composed of B-type Lamins (Lamin B1 and Lamin B3) plays a crucial role in nuclear remodeling during nuclear deformation in mammalian spermatogenesis [23,26]. To date, the study of Lamin B in crustacean spermiogenesis has not been reported. To explore the role of Lamin B in the spermiogenesis of *P. trituberculatus*, we prepared the antibody of Pt-Lamin B (Figure 4B). Localization of the Pt-Lamin B on the nuclear membrane in primary spermatocytes (Figure 5A1–A3′) indicates that Pt-Lamin B may be involved in maintaining the morphology of the spermatid nucleus and the stability of the nuclear membrane. Localization of the Pt-Lamin B on the nuclear membrane and on the cytoplasm in secondary spermatocytes (Figure 5B1–B3′) and early spermatids (Figure 5C1–C3′) indicates that a part of Pt-Lamin B may be transported from the nucleus to the cytoplasm. Localization of Pt-Lamin B on the nuclear membrane and on the cytoplasm in the middle spermatids (Figure 5D1–D3′), late spermatids (Figure 5E1–E3′), and mature sperm (Figure 5F1–F3′) indicates that Pt-Lamin B may be depolymerized in the spermatid nucleus and degraded out of the nucleus, promoting the deformation of the spermatid nucleus. Moreover, in our study, localization of the Pt-Lamin B on the gradually forming acrosome and on the nuclear membrane in spermatids (Figure 5) indicates that Pt-Lamin B may be involved in spermatid nucleus deformation and acrosome formation. This phenomenon has not been found in spermatogenesis in other species. Based on the above results, we speculate that Pt-Lamin B may depolymerize in the spermatid nucleus and export the nucleus into the cytoplasm to promote nuclear deformation. At the same time, localization of the Pt-Lamin B on the acrosome in middle spermatids and in late spermatids suggests that Pt-Lamin B may play a role in the formation of acrosomes in the middle and late stages and the late-fertilization process.

### 3.3. Dynein-1 Is Involved in the Nuclear Deformation Process of Spermiogenesis in P. trituberculatus

Dynein complex, as a microtubule-related regulatory protein, can regulate the localization of the cell nucleus by transporting some organelles [37]. DIC is a substantial connecting component of Dynein-1, which has the function of connecting DHC, DLIC, and DLC and regulating the Dynein complex [37].

To study the function of Dynein-1 in the deformation of the spermatid nucleus in *P. trituberculatus*, we carried out RNAi experiments at the individual level. The significant suppression of mRNA expression of the Dynein heavy chain *Pt-dhc* gene (Figure 8A) and unchanged expression of the Dynein light chain *Pt-km23* (Figure 8B) and *Pt-Lc8* (Figure 8C) genes and the Dynein-related regulatory factors *Pt-Nude* (Figure 8D) and *Pt-lis1* (Figure 8E) indicate that, as the intermediate chain of Dynein, *Pt-DIC* can affect the expression of Dynein heavy chain *Pt-dhc*. The Pt-DIC may have some influence on the function of the Dynein-1 heavy chain. Nonsignificant change in apoptosis-related genes indicates that interference with *Pt-DIC* expression has no effect on the apoptosis process of spermatogenic cells (Figure 8G–I).

The significance of the Pt-DIC signal was weakened in the *Pt-DIC* interference group during spermiogenesis in *P. trituberculatus* (Figure 9). The significant of the interference group Pt-DIC (Figure 9B) signal in the control group (Figure 9A) was weakened in the middle spermatids. The significant increase in the percentage of abnormal spermatid nuclei (Figure 9D) indicates that this may be due to the decreased expression of *Pt-DIC* inhibiting the function of Dynein-1, thus affecting the role of Dynein-1 in the process of spermatid nucleus deformation. The absence of the interference group (Figure 9B) and the control group (Figure 9A) in mature sperm is probably because the mature sperm in the interference group completed the nuclear-deformation process before interference.

### 3.4. Dynein-1 May Be Involved in the Transport of Pt-Lamin B in Middle and Late Spermatids

Given the partial transfer of the Pt-Lamin B signal to the perinuclear region to the cytoplasm position in the middle and late spermatids and its concentration in the acrosome (Figure 5), we speculate that Pt-Lamin B is gradually transported out of the nucleus during spermiogenesis and may be involved in the formation of acrosomes.

It has been reported that Dynein can promote nuclear membrane rupture by pulling the nuclear membrane and related proteins [47]. In addition, Dynein can mediate the transport of nuclear membrane residues containing Lamins along microtubules after the decomposition of the nuclear envelope during mitosis [48]. Is Pt-Lamin B, after the outing of the nucleus, also a transport substrate for Dynein-1? We found that Pt-Lamin B and Pt-DIC were colocalized at different stages of spermatogenesis in *P. trituberculatus* via immunofluorescence (Figure 6A). The protein interaction between Pt-Lamin B and Pt-DIC was further confirmed by coimmunoprecipitation (Figure 6C).

The expression of Pt-DIC decreased, and colocalization of Pt-Lamin B and Pt-DIC weakened in early spermatids (Figure 6A,B), which indicates that the nuclear-deformation process of spermiogenesis was affected. The colocalization of the Pt-Lamin B signal and Pt-DIC signal was weakened in the middle spermatid of the interference group, and the distribution of the Pt-Lamin B signal and Pt-DIC signal was changed in the acrosome, indicating that interference with *Pt-DIC* would affect the formation of the acrosome. The significance of the Pt-Lamin B signal and Pt-DIC signal was weakened in mature sperm of the interference group, indicating that interference with *Pt*-*DIC* would affect the localization of Pt-Lamin B.

In conclusion, the interference of Pt-DIC function will affect the normal distribution of Pt-Lamin B in spermatids, thus affecting the transport and normal function of Pt-Lamin B. Therefore, we speculate that Dynein-1 may be involved in the spermiogenesis of *P. trituberculatus* by mediating the transport of Lamin B.

## 4. Materials and Methods

### 4.1. Animals, Dissection, and Sample Preparation

Twelve adult male *P. trituberculatus* (80–100 g) used for the experiments were purchased from Shi-style Aquaculture Limited Company in Xianxiang Town, Ningbo, Zhejiang Province, China. *P. trituberculatus* were anesthetized on ice and dissected. The testes, vas deferens, hepatopancreas, heart, gills, and muscles were separated out. The isolated tissues were rapidly immersed in liquid nitrogen and stored at −80 °C. To make frozen sections of testes tissue, some of the above dissected testes tissues were fixed in an enzyme-free tube containing 4% PFA-PBS (paraformaldehyde-phosphate-buffered saline) (Beyotime, Shanghai, China) fixative for 2 h. The testes tissues were rinsed with 1× PBS (Beyotime, Shanghai, China) repeatedly 3 times and then immersed in 0.5 mol/L sucrose solution at 4 °C overnight. The fixed tissues were embedded in O.C.T complex (SAKURA, St. Torrance, CA, USA), frozen solidified to white-colored at −20 °C, and then stored at −80 °C. The experimental animal of this study was *P. trituberculatus*, a kind of crab, which is an invertebrate. In China, crabs do not require ethical approval for experiments.

### 4.2. RNA Extraction and cDNA Reverse Transcription

RNA was extracted from testes tissues isolated from *P. trituberculatus* using TRIzol reagent (Tiangen Biotech, Beijing, China). Reverse transcription was performed using the PrimeScript^®^ RT kit (Takara, Dalian, China) for cloning intermediate fragment sequences. The 3′ and 5′ fragment cDNAs were synthesized from *P. trituberculatus* isolated testes tissue using SMARTer RACE 5′/3′ reagent (Takara, Dalian, China). The obtained reverse-transcription products (cDNA) were stored at −80 °C.

### 4.3. Full-Length Cloning of Pt-Lamin B cDNA

The mRNA sequence of *Penaeus japonicus* Lamin Dm0 like (XM_043029302.1) was downloaded through the National Bioinformation Technology Center (https://www.ncbi.nlm.nih.gov, NCBI; accessed on 10 March 2022) as a query sequence for BLAST (Nucleotide BLAST: Search nucleotide databases using a nucleotide query (nih.gov); accessed on 10 March 2022) through NCBI *P. trituberculatus* gene library to obtain a partial mRNA sequence (NC_059256.1) of putative *P. trituberculatus Lamin B*. Primer Premier 5.0 (Premier Biosoft International, Palo Alto, CA, USA) software was used to design specific primers for cloning intermediate fragment of the mRNA sequence of *Pt-Lamin B* (Table 2). Amplification was performed using Touch down-PCR (TD-PCR) (Eppendorf, USA) mode: 94 °C for 5 min; 8 cycles of 94 °C for 30 s, 62–58.5 °C for 30 s (decreased by 0.5 °C/cycle), and 72 °C for 2 min 30 s; 27 cycles of 94 °C for 30 s, 58 °C for 30 s, and 72 °C for 2 min 30 s; and 72 °C for 10 min for the final extension. The PCR products were identified by 1.0% agarose gel electrophoresis. Following the instructions, a Quick-type DNA Gel Extraction Kit (BioTeke, Beijing, China) was used to recover the expected target bands to obtain DNA fragments, which were then ligated to the vector pMD-19T (Takara, Beijing, China), transformed into *Escherichia coli* DH5α-competent cells (AngYuBio, Shanghai, China), grown in the bacterial broth, and subsequently sent to Zhejiang Youkang Ltd., Yongkang, China for sequencing. Specific primers were designed for the obtained intermediate fragments for cloning 5′ and 3′ fragments (Table 2) using Primer Premier 5.0 software. Amplification was cloned by TD-PCR: 94 °C for 5 min; 8 cycles of 94 °C for 30 s, 71–67.5 °C for 30 s (decreased by 0.5 °C/cycle), and 72 °C for 3 min; 27 cycles of 94 °C for 30 s, 66 °C for 30 s, and 72 °C for 10 min for the final extension. The PCR products were performed as described above and sent for sequencing. The full-length cDNA sequence of *Pt-Lamin B* was obtained by splicing the correct fragment sequences according to the NCBI sequence in Vector NTI 11.5.1 (Invitrogen, Waltham, MA, USA) software.

### 4.4. Sequence Alignment, Phylogenetic Tree Building and Analysis, and Protein Structure PREDICTION

We downloaded 24 amino acid sequences of Lamin B from 7 vertebrate and 7 invertebrate species (Table 3). The amino acid sequences of Lamin B proteins were compared using Vector NTI 11.5.1 (Invitrogen, USA) software. The phylogenetic tree of species Lamin B proteins was constructed using MEGA 5.1 (Informar Technologies, Altamor Drive, LA, USA) software. The protein molecular weight was predicted using an online tool (http://www.biosoft.net/sms/; accessed on 10 April 2022). The isoelectric point was predicted using an online tool (https://web.expasy.org/compute_pi/; accessed on 10 April 2022). The secondary structure of the Lamin B protein was predicted using online tools (https://www.ncbi.nlm.nih.gov/Structure/cdd/wrpsb.cgi, http://bioinf.cs.ucl.ac.uk/psipred/; accessed on 10 April 2022). The nuclear localization signal was predicted using online tools (https://www.genscript.com/tools/psort, http://www.moseslab.csb.utoronto.ca/NLStradamus/; accessed on 10 April 2022)), and the tertiary structure of Lamin B was predicted using an online tool (https://zhanggroup.org//I-TASSER/; accessed on 7 April 2022)).

### 4.5. RNA Interference of Pt-DIC

Primer Premier 5.0 software was used to design a pair of *Pt-DIC*-specific primers as interference template gene fragments and another pair of specific primers in the interference template fragments. The sequences of the T7 promoter were added to the second pair of specific primers (Table 2). The PCR product of the interference template fragment was used as cDNA for PCR. After electrophoresis of their PCR products, gel recovery was carried out. Primers were amplified using PCR to obtain DNA fragments, which were used as the template for dsRNA synthesis. The T7 RiboMAX Express RNAi System (Promega, Madison, WI, USA) was used to synthesize dsRNA according to the instructions and performed gel electrophoresis detection. A NanoDrop2000 (Thermo Fisher Scientific, Waltham, MA, USA) was used to measure the concentration of dsRNA using 1× PBS diluted to 1.5 μg/μL. *P. trituberculatus* was temporarily raised for one week, and an RNA interference test was conducted after its vital signs were stable. Each crab in the experimental group (six crabs) was injected with 1 μL/g dsRNA at the bottom of the fourth swimming foot, and each crab in the control group (six crabs) was injected with 1 μL/g 1× PBS at the bottom of the fourth swimming foot. Injection was performed five times in both the experimental and control groups, once every 48 h. After the last injection for more than 24 h, all *P. trituberculatus* were dissected on ice in turn. The testes tissue was made into frozen sections for subsequent immunofluorescence experiments. The RNA of each crab testes was extracted and then reverse-transcribed for qPCR.

### 4.6. qPCR

Primer Premier 5.0 software was used to design specific primers for *Pt-DIC*, *Pt-Lamin B*, and *Pt-LC8* (which have been cloned and uploaded to NCBI, GenBank number OQ506131). *Pt-km23* (GenBank number MZ277768), *Pt-lis1* (GenBank number OL841528), *Pt-Nude* (GenBank number OQ556853), *Pt-dhc*, *p53*, *caspase-3*, *Pt-bcl-2*, and quantitative primer references for *GAPDH* (Table 2) [5,49]. Then, 2× RealStar Green Fast Mixture kits (Genstar, Beijing, China) were used for qPCR to detect gene expression levels. A total of 12 samples were used, and the experiments were performed in three parallels. The reaction system was 8 μL of cDNA template (1 μL cDNA diluted 25 times with purified water), 1 μL of each primer, and 10 μL of 2× RealStar Fast SYBR qPCR Mix. The qPCR program was completed with predenaturation at 95 °C for 2 min and 40 cycles (denaturation at 95 °C for 15 s, annealing at 60 °C for 30 s, and extension at 72 °C for 30 s). PBS group was used as a control group. The interference efficiency of *Pt-DIC* mRNA and expression of its related genes (*Pt-Lamin B*, *Pt-LC8*, *Pt-km23*, *Pt-lis1*, *Pt-Nude Pt-dhc*, *p53*, *caspase-3*, and *Pt-bcl-2*) after the interference was analyzed using comparative ΔΔCt method [49].

### 4.7. Antibodies

#### 4.7.1. Prokaryotic Expression

The software Primer Premier 5 was used to design a pair of *Pt-Lamin B*-specific primers as antigen fragments. The *Pt-Lamin B* antigen fragment was 324 bp (encoding 108 amino acids, approximately 12.13 kDa). *Bam*H I and *Xho* I (Takara, Beijing, China) restriction endonuclease sites were added at both ends of the Pt-Lamin B antigen fragment. PCR reaction was performed with the primers of LaminYH-F and LaminYH-R (Table 2). Amplification was cloned using the following PCR mode: 94 °C for 5 min (35 cycles of 94 °C for 30 s, 54.5 °C for 30 s, and 72 °C for 50 s) and 72 °C for 10 min. The recovered PCR product and the plasmid pET-28a (+) (Takara, Beijing, China) were subjected to a double-enzyme digestion reaction followed by ligation and transformation to *E. coli* DH5α competent cells (AngYuBio, Shanghai, China). After 4 h of shaking, the appropriate amount of bacterial liquid was smeared on Kana+ solid medium overnight culture. Then, a single colony was selected for PCR to pick the positive bacteria, and the plasmid was extracted by a Plasmid Extraction Mini Kit (Solarbio, Beijing, China). After obtaining the correct Lamin B recombinant plasmid after the sequencing test, it was transferred into the Transetta strain (empty pET-28a plasma was used as a control) and inoculated in 300 mL Kana+liquid medium for mass amplification and cultured (37 °C, 180 rpm) with protein expression induced with 1 mM IPTG. The His-tagged protein purification kit (Beyotime, Shanghai, China) was used to purify the corresponding protein according to the instructions. The purified fusion protein (purity > 85%, concentration of 1 mg/mL, total of 7 mg) was injected into New Zealand white rabbits several times, and the final serum was a Pt-Lamin B polyclonal rabbit antibody. Two male New Zealand white rabbits were purchased from Jianfei Experimental Rabbit Farm, Simen Town, Yuyao, Ningbo (SCXK (Zhejiang) 2022-0002) and fed in separate cages at the Experimental Animal Center of Ningbo University (SYXK (Zhejiang) 2019-0005). The number of the animal ethics application project for this research is 11709. Animal experiment of immunizing New Zealand white rabbits was approved by the Animal Ethics and Welfare Committee of Ningbo University (approval number NBU20220155).

#### 4.7.2. Western Blot (Verification of Pt-Lamin B Antibody)

Testes tissues were disrupted in the mixture of 1 mL of RIPA (radio-immunoprecipitation assay) buffer (with 10 μL of PSMF) (Beyotime, Shanghai, China) was used to extract the total protein from the testes of *P. trituberculatus* (50–100 mg), and the protein concentration was measured and calculated with an enzyme marker. Twelve percent gel electrophoresis (SDS–PAGE) was used to separate and transfer proteins from testes tissue to PVDF membranes activated by methanol (Bio-Rad, Nobel Drive Hercules, CA, USA). After the membranes were transferred, they were washed with 1× TBST for 5 min. The transferred PVDF membranes were placed in 5% skim milk powder sealing solution at room temperature for 2 h and then incubated with Lamin B antibody (1:200 dilution) at 4 °C overnight. After removing the PVDF membrane, the membrane was rinsed with 1× TBST four times for 15 min each time. Then, goat anti-rabbit IgG (1:1500 dilution) labeled with horseradish peroxidase (HRP) was added to the membrane, and the membrane was incubated at 37 °C for 2 h. The membrane was removed, and the membrane was rinsed with 1× TBST 4 times for 15 min each time. Finally, the developer was prepared in proportion and added uniformly to the PVDF film for development and chemiluminescence imaging (Tanon 5200; Shanghai, China). The antibody against *P. trituberculatus* DIC used in this study was homemade in the laboratory and has been validated [5].

### 4.8. Immunofluorescence

The testes of *P. trituberculatus* were cut into 5 μm slices on adhesive microscope slides (Citotest, Jiangsu, China) using a frozen sectioning machine and stored at −80 °C. Before immunofluorescence, the slices were removed and dried at room temperature for approximately 10 min and soaked with 0.3% PBST for 20 min. Then, sufficient 5% BSA-PBS was added, and the sections were attached to a sealing film and incubated at 37 °C for 1.5 h. After the blocking solution was discarded, the sections were incubated with a sufficient amount of primary antibody (Lamin B rabbit antibody 1:50/DIC mouse antibody 1:50 in PBST), covered with sealing film, and incubated overnight in a wet box at 4 °C. In the negative control group, antibody diluent was added instead of the primary antibody. The following operations were performed in the dark. The sections were washed with 0.1% PBST 3 times for 10 min each time. After washing, the slices were removed and incubated with Fluor 555-labeled goat anti-rabbit IgG (H+L) (Beyotime, Shanghai, China) and Alexa Fluor 488-labeled donkey anti-mouse IgG (H+L) (Beyotime, Shanghai, China) for 60 min. The slices were washed with 0.1% PBST 6 times for 15 min each, and then DAPI (Beyotime, Shanghai, China) was added to stain the sections at room temperature for 5–10 min. The sections were rinsed with 0.1% PBST, and 10 μL of antifade mounting medium (Beyotime, Shanghai, China) was added. The sections were covered with cover glass, sealed with nail polish, and placed at 4 °C overnight. The cells were observed under a Zeiss laser scanning confocal microscope (LSM880, Carl Zeiss, Germany).

### 4.9. Coimmunoprecipitation (Co-IP)

Testes tissues were disrupted in the mixture of Western and IP cell lysates (Beyotime, Shanghai, China) and protease inhibitor. The tissue homogenate was centrifuged at 14,000 rpm for 5 min (4 °C). We collected the supernatant and measured the total protein concentration using BCA Protein Assay Kit (Beyotime, Dalian, China). A total of 20 μL of common serum whose antiserum species were the same as those used in immunoprecipitation and 40 μL of fully resuspended Protein A+G Agarose (Beyotime, Shanghai, China) were added to 800 μL of extracted spermatid protein solution and slowly shaken at 4 °C for 1.5 h. Subsequently, the samples were centrifuged at 12,000× *g* for 1 min at 4 °C, and the supernatant was transferred to a 1.5 mL sterile centrifuge tube. Then, 40 μL of antiserum was added to the upper clear section of the previous step and slowly shaken overnight at 4 °C. Then, 40 μL of fully resuspended Protein A+G Agarose was added to the solution overnight in the previous step, slowly shaken at 4 °C for 1.5 h, and centrifuged at 4 °C for 1 min at 12,000× *g*. The supernatant was discarded, and 1× PBS flushing sedimentation was used 8 times; each time, the dosage of 1× PBS was 700 μL. After washing and settling the sedimentation, the supernatant was discarded, 20 μL of 1× SDS–PAGE electrophoresis sample loading buffer was added, and sufficient resuspension was performed. The previous solution was placed in a 100 °C metal bath (Sangon Biotech, Shanghai, China) for 5 min and centrifuged at 12,000× *g* for 1 min, and then the supernatant was taken directly for Western blot detection of protein interactions.

### 4.10. Statistical Analysis

Experimental statistical data were presented as the mean ± standard deviation (SD). All analyses and graphs were performed using IBM SPSS Statistics 25.0 (SPSS Software, Chicago, IL, USA) and GraphPad Prism software 8.0.2 (GraphPad Software Inc., San Diego, CA, USA). All statistical data were analyzed with the use of one-way analysis of variance (ANOVA) to determine the significance of differences, followed by post hoc Tukey’s multiple comparison testing. A *p* value of less than 0.05 was considered significant. Statistical analysis data are shown in attached Supplementary File S1.

## 5. Conclusions

In this study, we obtained the full length of *Pt-Lamin B* from the testes of *P. trituberculatus* and performed bioinformatics analysis. It was found that Pt-Lamin B was highly conserved in evolution with invertebrates. The expression and distribution of Pt-Lamin B protein during spermatogenesis suggest that Pt-Lamin B is involved in nuclear deformation and acrosome formation. Dynein-1 and Pt-Lamin B proteins were colocalized in the middle spermatid of *P. trituberculatus*. Coimmunoprecipitation further demonstrated the interaction between Pt-DIC and Pt-Lamin B in the testes of *P. trituberculatus*. It was demonstrated that the Pt-DIC protein and Pt-Lamin B protein were involved in the spermiogenesis process of *P. trituberculatus*. After *Pt-DIC* interference, the distribution of Pt-DIC and Pt-Lamin B in spermatogenesis was abnormal, and the colocalization weakened. The abnormal proportion of spermatid nucleus deformation in the middle stage significantly increased. This indicates that Pt-DIC could affect the normal distribution and function of Pt-Lamin B. In conclusion, both Pt-DIC and Pt-Lamin B are involved in the spermiogenesis of *P. trituberculatus*. Dynein-1 may participate in the spermiogenesis of *P. trituberculatus* by mediating the transport of Lamin B after nuclear export via Pt-DIC.

## Figures and Tables

**Table 1:** The amino acid sequence identity comparing *P. trituberculatus* Lamin B and other different Lamin B proteins.

Species	Identity
*Homo sapiens*	28.0%
*Bos taurus*	27.8%
*Gallus gallus*	26.4%
*Mus musculus*	27.5%
*Xenopus tropicals*	22.3%
*Danio rerio*	28.6%
*Salmo salar*	28.2%
*Drosophila melanogaster*	39.1%
*Procambarus clarkii*	66.1%
*Penaeus vannamei*	43.8%
*Penaeus monodon*	63.7%
*Penaeus japonicus*	64.2%
*Penaeus chinensis*	63.7%
*Caenorhabditis elegans*	26.6%

**Table 2:** The primer sequences used in the present study.

Primer	Sequence (5′→3′)	Purpose
Lamin B-F	GTGTTTGAATGACAGGCTGGC	PCR
Lamin B-R	GAGCCGCAAATCCTCGTTAG	PCR
3′LaminB-F1	CAACTGGAACTGGAGAGCAACCGACT	3′RACE
3′Lamin B-F2	TTAGTAAGGAGGTTGTGAGCATGAAGGG	3′RACE
5′Lamin B-R1	CGAGAAGTTTCCGAGCGTCAGAGAGTT	5′RACE
5′Lamin B-R2	TTGAGTCGGTTGCTCTCCAGTTCCA	5′RACE
DIC-mbF	CGACGGGCACGGTTACTAT	RNAi
DIC-mbR	AGGCTTCGTTTCCTTGACA	RNAi
T7dsDICF	**TAATACGACTCACTATAGGG**GGATGGAGAGGCTAACGA	Positive template
dsDICR	ACATCTTGCCATCAGTGCT	RNAi
dsDICF	GGATGGAGAGGCTAACGA	RNAi
T7dsDICR	**TAATACGACTCACTATAGGG**ACATCTTGCCATCAGTGCT	Reverse template
LaminYH-F	CGCGGATCCCAACTGGAACTGGAGAGCAA	Prokaryotic expression
LaminYH-R	CGCGGATCCCAACTGGAACTGGAGAGCAA	Prokaryotic expression
DIC-F	TATGAGGTAGCCTGGTCCCC	qPCR
DIC-R	GCTGGCCAGAGTTAGTCCAA	qPCR
P53-F	GGGTAACGCCATGAACGAGA	qPCR
P53-R	GCTGCATCTCCGTGTGTTTC	qPCR
Caspase3-F	TCACAGATTGACAAAGAGCGG	qPCR
Caspase3-R	TCCTCAGGTCAGTAGTGGAAATG	qPCR
km23-F	TGTCAGCAGAGGTTGAAGAAA	qPCR
km23-R	AGGAATGTGAGGTCGTTGGT	qPCR
Lamin B-qF	TTACTCGCCGAACTCAAGGA	qPCR
Lamin B-qR	TGAAGTCCTCGTAGTAGCAGCA	qPCR
BCL2-F	AGCTTACAACTGGATGCGCT	qPCR
BCL2-R	TCGAGAGTGATTTAGGCGGC	qPCR
LC8-F	ACATCGCCGCCTACATCAA	qPCR
LC8-R	GGGGCACACTTAGCCACTCT	qPCR
Lis1-F	TGCCACAGATAACCGAAAGC	qPCR
Lis1-R	TTGTCGTGACCCACCAGAGA	qPCR
Nude-F	AGGAGTTTCAGAGTGGCAGCA	qPCR
Nude-R	TTTTGTAGCCTTTCCAGACGC	qPCR
GAPDHF	TGAGGTGAAGGTAGAGGAT	qPCR
GAPDHR	CCAGTGAAGTGAGCAGAG	qPCR

Note: bold bases are the added T7 promoter.

**Table 3:** Species and their GenBank numbers of *Lamin B*.

Specie	Protein	GenBank
*Homo sapiens*	Lamin B1/B2	NP_005564.1/NP_116126.3
*Bos taurus*	Lamin B1/B2	NP_001096765.1/NP_001263282.1
*Gallus gallus*	Lamin B1/B2	NP_990617.2/NP_990616.2
*Mus musculus*	Lamin B1/B2	NP_034851.2/NP_034852.3
*Danio rerio*	Lamin B1/B2	NP_694504.2/NP_571077.2
*Salmo salar*	Lamin B1/B2	XP_013999495.2/XP_013979108.1
*Xenopus tropicals*	Lamin B1/B1S	NP_989198.1/NP_001081547.1
*Xenopus tropicals*	Lamin B1L/B3	NP_001080053.1/NP_001076823.1
*Xenopus tropicals*	Lamin B3L	NP_001081545.1
*Drosophila melanogaster*	Lamin Dm0	NP_476616.1
*Procambarus clarkii*	Lamin Dm0 like	NP_001289961.1
*Penaeus vannamei*	Lamin Dm0 like	XP_027220822.1
*Penaeus monodon*	Lamin Dm0 like	XP_037794519.1
*Penaeus japonicus*	Lamin Dm0 like	XP_042885236.1
*Penaeus chinensis*	Lamin Dm0 like	XP_047494326.1
*Caenorhabditis elegans*	Lamin 1	NP_492371.1

## Data Availability

Data are contained within the article and Supplementary Materials.

## Supplementary Materials

The following supporting information can be downloaded at: https://www.mdpi.com/article/10.3390/ijms25010112/s1.
